# Correction: Gurevitz, M.; Leisman, G. Factors in Infancy That May Predict Autism Spectrum Disorder. *Brain Sci.* 2023, *13,* 1374

**DOI:** 10.3390/brainsci14030191

**Published:** 2024-02-20

**Authors:** Mina Gurevitz, Gerry Leisman

**Affiliations:** 1 Well Baby Clinic Physician, Maccabi Health Services, Herzliya 4649713, Israel; mina.gurevich@gmail.com; 2 Movement and Fetal Cognition Laboratory, Department of Physical Therapy, University of Haifa, Haifa 3498838, Israel; 3 Department of Neurology, University of the Medical Sciences of Havana, Havana 11600, Cuba

There was an error in the original publication [1]. The published study is based on the derivation of detailed information from the Maccabi Health Services (MHS). The MHS are a state-mandated health maintenance organization in Israel with 2.5 million enrollees, servicing 26% of Israel’s population. The electronic medical records integrate data from the Maccabi Health Services’ central laboratory, medication prescriptions, records of purchases within the MHS pharmacy network, consultations, hospitalizations, procedures, and sociodemographic information. The database also integrates socioeconomic statuses, provided by the Central Statistics Bureau. As a result, (a) access to the database is not permitted for reasons including confidentiality, but (b) the manipulation of the data from the database with the appropriate ethical controls is permitted, although it cannot be released, and (c) the biostatistics division of the Maccabi HMO can work with individuals having questions about the aggregated data found in the database. Ms. Liat Lev-Shalem serves as a “point-of-contact” for data access-related issues. (d) The presence of the following statement at the end of the paper, “The datasets for this study can be found in ResearchGate and will be available on request”, was, through oversight, missed in the final proofreading of the manuscript.

A correction has been made to the Data Availability section at the end of the paper and should read as follows:

“The datasets for this study are part of the Big Data Repository of the Maccabi HMO; questions about the database and the data from the current study may be addressed to Liat Lev-Shalem, member of the MAROM program.”

The authors state that the scientific conclusions in the published study are unaffected. This correction was approved by the Academic Editor. The original publication has also been updated.
